# Commentary: Beyond 10-year risk: A cost-effectiveness analysis of statins for the primary prevention of cardiovascular disease

**DOI:** 10.3389/fcvm.2022.916695

**Published:** 2022-07-22

**Authors:** Aditya Shah, Kamal Sharma, Shalin Rawal, Rhea Sisodia, Parjanya Bhatt, Cleris Christian, Ashwati Konat

**Affiliations:** ^1^B.J. Medical College and Civil Hospital, Ahmedabad, India; ^2^SAL Hospital, Ahmedabad, India; ^3^Department of Zoology, Biomedical Technology and Human Genetics, Gujarat University, Ahmedabad, India

**Keywords:** statins, cost-effectiveness, absolute risk reduction (ARR), incremental cost-effective ratio, generic drug

## Introduction

Cardiovascular diseases have infamously been the frontrunners in terms of morbidity and mortality. These diseases have a physical, mental and economic toll on the patients. This has prompted researchers to carry out cost-effectiveness analyses for preventive therapies for cardiovascular diseases. One of the best preventive modalities is cholesterol management using Statins—a wonder drug which decreases cholesterol synthesis by inhibiting the Hydroxymethylglutaryl-CoA (HMG-CoA) reductase enzyme (1, 2).

Various studies such as the West of Scotland Coronary Prevention Study (WOSCOPS) have proved the cost-effectiveness of statins in the primary prevention of cardiovascular diseases (3). Amongst this, Kohli-Lynch et al. carried out research which shed light on the cost-effectiveness of various statin prioritization strategies. Even though the study was successful in evaluating the strategies, there were a few factors such as the possible drug-drug interactions and the reality of generic pricing which were overlooked. This commentary aims at pointing out how the researchers considered an ideal scenario which could have impacted their analyses.

The study evaluates the cost-effectiveness of statins in terms of monetary expenses and their impact on quality of life (QOL) (4). This was done by calculating the incremental cost-effectiveness ratio (ICER) per quality-adjusted life-year (QALY) gained. In simpler terms, they calculated how much a patient must pay for each additional year of life acquired as a result of therapy. It was considered cost-effective if the value was less than the country's average willingness-to-pay threshold.

**Figure 1:**
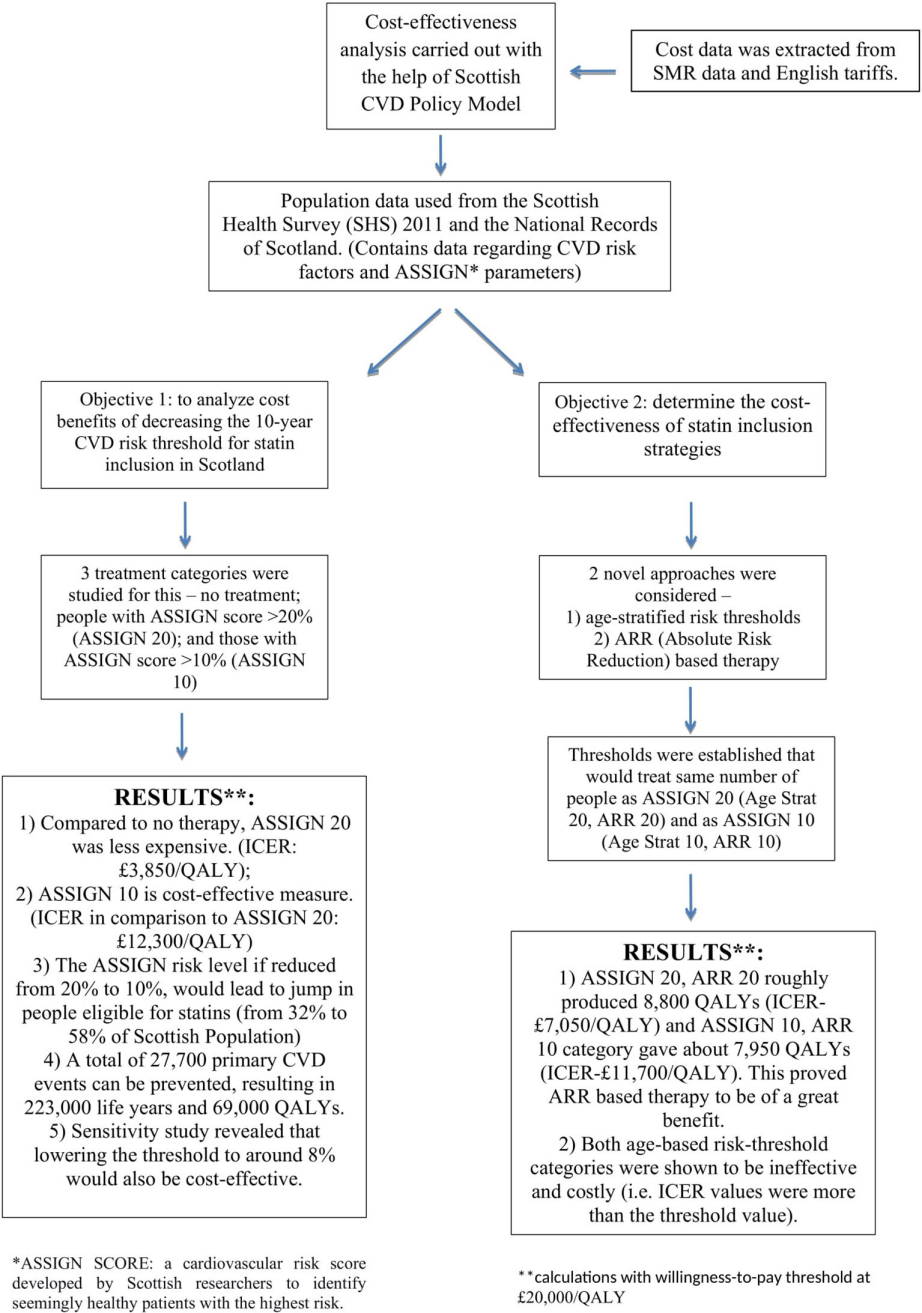
Study design and result.

Kohli-Lynch et al. employed the “Scottish CVD Policy model” and ran simulations to calculate the cost-effectiveness of the 3 prioritization strategies-−10-year risk threshold, Age-stratified risk thresholds and Absolute risk reduction (ARR) guided therapy (Figure 1).

The outcome of the study was measured in terms of the incremental cost/QALY gained for different treatment policies and primary CVD events prevented. The research was carried out from the standpoint of the health sector, and a plan was labeled cost-effective if its incremental cost was < £20,000/QALY, the usual Scottish criterion (5, 6).

## Discussion

The first goal of the study was to find the cost-effectiveness of lowering the risk threshold and increasing the statin eligibility in population aged ≥40 years. Even though the results show it to be cost-effective, it overlooks the impact of the drug-drug interactions of statins with other medications. For example, if we consider a case of a patient with HIV-AIDS, generic statins such as Atorvastatin and Simvastatin are contraindicated to be given with antiviral protease inhibitors (7). The safest options are Pitavastatin and Fluvastatin (8) which are costlier and not sold in generic form. This negatively impacts the cost-effectiveness analyses.

The second goal was to find the cost-effectiveness of statin prioritization strategies. The 10-year CVD risk calculation approachdoesn't consider non-CVD mortality due to a common risk factor like age. This overestimates the benefits of the therapy and calls for the need to go beyond 10-year risk scoring.

Age-stratified risk threshold-based policy is considered beneficial mainly because it helps in treating younger individuals with higher risk. However, the results reveal it to be expensive. According to the researchers, longitudinal risk factor data is necessary for an accurate evaluation, but they were unable to find such data in the context of the Scottish population. The absence of such crucial data could have led to an underestimation of the benefits of the therapy. A proper study should be designed to discover the potential of this policy which can be useful in cases where Absolute Risk Reduction (ARR)-based therapy is unfavorable—such as treating large number of people with high pill-taking disutility.

Out of all the policies, the ARR-based strategy proved to be superior in the majority of model runs and sensitivity analyses. When all treatment options were evaluated collectively, ARR was favorable 88% of the time. ARR-based therapy was based on baseline 10-year CVD risk and non-HDL levels. Other studies have used strategies which include LDL-C levels. But Kohli-Lynch et al. have done brilliant work by switching it with non-HDL-C levels. The latter can be easily calculated and is superior in predicting CVS mortality in comparison to LDL-C levels (9).

Consideration of important external factors such as non-compliance with medication, inflation rates, country health policies and risk of statins-induced diabetes and the associated costs, highlight the completeness of the model.

An important factor that draws our attention is that the overall population disproportionately consisted of females, as established CVD cases were excluded. This makes us question if the results were gender-biased up to a certain extent. Since ARR-based therapy covers high-risk individuals, including males, it can be used to make the study more inclusive and less biased.

The entire cost-effectiveness analysis of the statins was given an impetus by the generic pricing of it. However, we cannot be blindsided by the scams and conspiracies that surround the manufacturing of generic drugs. Several pharmaceutical companies have been caught defrauding the government by fixing the price of generic drugs (10, 11).

Another point that deserves a discussion is the effectiveness of generic drugs. Many practitioners have disagreed with the use of generic drugs and questioned the clinical equivalency with their newer medications (12). Even patients have been skeptical about the use of generic drugs and have raised doubts about their safety and quality (13).

Such factors can impact the usage of generic drugs and the cost-effectiveness analyses.

## Conclusion

Kohli-Lynch et al. study carries out well-rounded research in comparing the novel approaches. The impact of generic drugs was predicted by early researchers and they tapped this idea to explore other unanswered questions. A similar model can be used by other countries to calculate the cost-effectiveness of increasing statin eligibility. Since different countries have different economic conditions, it would pave the way for the formulation of newer cost-effect statin prioritization strategies. This will further help us in reducing the morbidity and mortality due to cardiovascular diseases.

## Author contributions

All authors listed have made a substantial, direct, and intellectual contribution to the work and approved it for publication.

## Conflict of interest

The authors declare that the research was conducted in the absence of any commercial or financial relationships that could be construed as a potential conflict of interest.

## Publisher's note

All claims expressed in this article are solely those of the authors and do not necessarily represent those of their affiliated organizations, or those of the publisher, the editors and the reviewers. Any product that may be evaluated in this article, or claim that may be made by its manufacturer, is not guaranteed or endorsed by the publisher.
